# Characterization and phylogenetic analysis of the first complete mitochondrial genome sequence of three *Artocarpus* species in Hainan Province

**DOI:** 10.3389/fpls.2025.1733932

**Published:** 2025-12-19

**Authors:** Huanwei Wang, Hongyan Fan, Yuhong Qin, Chunmei Wu, Ya Zhao, Norvienyeku Justice, Min Xiao, Shaoka Li, Weiguo Miao, Wenbo Liu

**Affiliations:** 1 Key Laboratory of Green Prevention and Control of Tropical Plant Diseases and Pests, Ministry of Education, School of Tropical Agriculture and Forestry, Hainan University, Haikou, China; 2 Danzhou Invasive Species Observation and Research Station of Hainan Province, Hainan University, Danzhou, China; 3 Tropical Fruit Trees Institute, Hainan Academy of Agricultural Sciences, Haikou, China; 4 Hainan Field Scientific Observation and Research Station for Tropical Fruit Trees; Haikou Tropical Fruit Tree Scientific Observation and Experimental Station, Ministry of Agriculture and Rural Affair, Haikou, China; 5 Hainan Key Laboratory of Tropical Fruit Trees Biology, Key Laboratory of Genetic Resources Evaluation and Utilization of Tropical Fruits and Vegetables (Co-construction by Ministry and Province), Ministry of Agriculture and Rural Affairs, Haikou, China

**Keywords:** Artocarpus, mitochondrial DNA, comparative genomics analysis, predicted RNA editing sites, phylogeny

## Abstract

The *Artocarpus* genus, belonging to the Moraceae family, exhibits various pharmacological and biological functions. However, the mitochondrial DNA (mtDNA) of *Artocarpus* species remains largely unexplored, which hampers our understanding of its phylogenetic classification as well as population identification. In this study, we completely sequenced and assembled the mtDNA of three *Artocarpus* species, including *Artocarpus heterophyllus*, *A. heterophyllus*(*R*), and *A. integer*. Three *Artocarpus* species exhibited highly similar mtDNA features, with mtDNA sizes of approximately 438,620 bp, consisting of six contigs, and included 32 different protein-coding genes (PCGs). The codon usage analysis demonstrated that Leucine and Serine were the most preferred amino acids in three *Artocarpus* species. Furthermore, in three *Artocarpus* species mt genomes, 9 homologous fragments were found to transfer from the cp genome, which contain complete *psaB*, *psaA*, *ndhB* and *rps7* genes. Phylogenetic trees further reveal that three *Artocarpus* species are most closely related to *Ficus carica* and *Morus notabilis*. In summary, this study fills the gap in mitochondrial genome data within the *Artocarpus* genus and provides a theoretical foundation for further understanding the taxonomic classification within *Artocarpus* species.

## 1 Introduction

Mitochondrial DNA (mitogenomes) evolve independently from nuclear genomes, providing valuable insight into the evolutionary history of their host species (Seidl, 2024). Plant mitochondria can significantly influence various biological functions, including stress tolerance, growth vigor, and cytoplasmic male sterility (Guo et al., 2016; Kianian, 2016). To date, a total of 688 plant mitogenomes have been reported, the number less than the chloroplast genomes (n = 12,989) and plastid genomes (n = 1,718) (November 18, 2025, https://www.ncbi.nlm.nih.gov/datasets/organelle/). Plant mitochondria are recognized for their distinctive semi-autonomous genetic properties, including gene sequence transfer or loss, multiple RNA editing modifications, and multipartite genome arrangements (Handa, 2003; Broz, 2022a; Song et al., 2024). Among them, frequent recombination of repetitive sequences is a common feature in mitochondrial genomes, and extensive endosymbiotic gene transfer events regularly occur from chloroplasts to mitochondria (Keeling and Palmer, 2008; Bock, 2010; Broz, 2022a; Broz, 2022b). These processes drive plant mitogenome expansion and accelerated evolution (Prasad et al., 2022). Moreover, RNA editing events are prevalent in plant mitochondrial transcripts, and are essential for the generation of protein function and adaptive evolution (Ichinose and Sugita, 2017; Broz, 2022a). The frequency and type of these events are phylogenetically constrained among congeneric species, making them informative for elucidating evolutionary relationships (Zhang et al., 2024). In conclusion, plant mitogenomes have emerged as vital tool for species classification, evolutionary studies, and parentage tracing.

*Artocarpus heterophyllus*, commonly known as jackfruit, is an exotic species native to the Western Ghats of India (Prakash et al., 2009; Baliga et al., 2011; Almeida et al., 2022). It is widely cultivated the tropical regions, and serves as an important tropical commercial crop in Hainan Province of China (Kumar et al., 2022; Zhao et al., 2023b; Zhao et al., 2023a). Recent studies have demonstrated that *Artocarpus* species are valuable sources of enzyme inhibitors, antioxidants, resveratrol, cosmeceuticals, and other bioactive compounds (Borah et al., 2017; Liyanaarachchi et al., 2018; Liyanaararchchi et al., 2022). The genus *Artocarpus* (Moraceae) comprises more than 70 recognized species with taxonomically diverse members (Gardner et al., 2021). *A. heterophyllus*, *A. heterophyllus* (R) and *A. integer* are the three major varieties primarily cultivated in Hainan, with an annual output value of up to 2 billion. *A*. *heterophyllus* is a treasure trace of medicinal potential, as its fruits, bark, leaves, and roots can be used in the treatment of various conditions, such as anemia, asthma, dermatosis, diarrhea, and cough (Muyonga and Nansereko, 2021; Baliga et al., 2011; Kumar and Mishra, 2022). *A. heterophyllus* (R), a red-fleshed jackfruit, is a new dry-bulb cultivar selected from natural seedlings of Thai jackfruit with higher economic value than conventional varieties (Zehuai et al., 2012). In addition, chempedak (*Artocarpus integer Merr*), known as Champada in Thai, is indigenous to southeast Asia. The fruit is morphologically similar to *A. heterophyllus*, though smaller in size and characterized by a softer texture. Upon ripening, it exhibits a flavor profile reminiscent of durian, accompanied by a potent and distinctive aroma (Buttara et al., 2014). Given their substantial economic and medical significance, it is imperative to investigate their evolutionary history. Currently, comparative studies of *A*. *heterophyllus* focus solely on chloroplast (cp) genomics (Liu et al., 2018), and nuclear genomes (Lin X et al., 2022). However, there have been no reports on the mitochondrial genomics of *Artocarpus* species. Research demonstrates that plant mitochondrial genomes have significant potential for the development of molecular markers and taxonomic classification, serving as a key resource in advancing research on plant population genetics and evolutionary biology (Wang et al., 2024). Furthermore, comparative analysis of mitochondrial genomes across several species within the same genus enables a comprehensive understanding of their structural organization and genomic diversity, offering valuable insights into mitogenome evolution at the genus level (Soe et al., 2025).

Until now, only five mitochondrial genomes from species within the Moraceae family have been successfully assembled and deposited in the National Center for Biotechnology Information (NCBI) database (as of November 7, 2025), including those of *Ficus carica*, *Morus notabilis*, *R. laevigata*, *R. hybrid cultivar*, and *R. chinensis*. Notably, no *Artocarpus* mitochondrial genomes have been published accessible, that hampers our understanding of its phylogenetic classification as well as population identification. In this study, we first sequenced and assembled the complete mitogenomes of three *Artocarpus* species and further analyze their structural characteristics, including gene arrangement, repeat sequences, codon preferences, Ka/Ks value, gene loss, phylogenetic position, sequence transfer between chloroplast and mitochondrial DNA, and RNA editing sites. This research will enhance our understanding of the complex evolutionary features of *Artocarpus* species, and lay a theoretical foundation for their evolution.

## 2 Material and methods

### 2.1 Assembly and annotation of mitochondrial DNA

The healthy leaves of *A. heterophyllus*, *A. heterophyllus*(R), and *A. integer* were collected from jackfruit orchard in Danzhou City, Hainan Province (109°49′E, 19°50′N). Each sample was immediately frozen in liquid nitrogen, and then stored at −80 °C. High-quality genomic DNA extractions were carried out from young leaves using a modified CTAB procedure (Porebski et al., 1997). The quality and concentration of DNA samples were evaluated using agarose gel electrophoresis and a NanoDrop spectrophotometer (Thermo Fisher Scientific, CA, USA). High-quality DNA samples were selected for library construction and sequencing. A 15-kb library was prepared using the SMRTbell Express Template Preparation Kit 2.0 (Pacific Biosciences, Menlo Park, CA, USA), following a standardized workflow that included DNA shearing, removal of single-stranded overhangs, DNA damage repair, end repair with A-tailing, adapter ligation, multiple rounds of AMPure PB bead purification (1X) after each critical step, nuclease treatment, size selection, quality control, primer annealing, polymerase binding, and final sequencing. After quality control (>Q20), the SMRTbell library was sequenced on the PacBio Revio platform (Pacific Biosciences, CA, USA) by Shenzhen Huitong Biotechnology Co., Ltd.

Each sample was sequenced using the PacBio Revio platform to generate at least 5 G of high-quality HiFi reads. Then, PMAT 1.5.3 was used to assemble the HiFi reads, with the genome size set at 1G and other parameters left at their default settings (Bi et al., 2024). The mitochondrial graph was extracted from the HiFi read assembly graph, resulting in a complete mitochondrial genome. Contig connections were validated using Bandage alignment (Wick et al., 2015), and the contig was circularized based on sequencing coverage. Three mitochondrial DNA were annotated utilizing Mitofy (Alverson et al., 2010) and MFannot (Gautheret and Lambert, 2001). After annotation, the OGDRAW program was used to draw the mitochondrial genome circular map (Greiner et al., 2019).

### 2.2 Repeat sequences and chloroplast-to-mitochondrial fragment analysis

Simple sequence repeats (SSRs), also known as microsatellites, were identified using the MISA v1.0 software with the following parameters: 1–10, 2–5, 3–4, 4–3, 5–3, and 6–3 (Benson, 1999). Tandem repeats were detected using TRF 4.09 (v4.09) under the parameter settings: 2 7 7 80 10 50 2000 -f -d -m (Benson, 1999). REPuter was employed to characterize dispersed repeats of ≥ 30 bp, including forward, reverse, palindromic, and complementary repeats (Iriarte et al., 2021). The resulting repeat patterns were visualized using Circos v0.69-6 (Zhang et al., 2013). The assembled complete chloroplast genome sequence of *A. heterophyllus*, *A. heterophyllus*(R), and *A. integer* has been submitted to NCBI (Accession number: PQ835410, PQ835412, PQ835411). Homologous fragments between the chloroplast and mitochondrial DNA of three *Artocarpus* species were identified using BLAST v2.2.26 (default parameters) (Altschul et al., 1990), and the results were visualized using Circos v0.69-5 (Zhang et al., 2013).

### 2.3 Codon usage identification and prediction of RNA editing sites

Perl scripts was used to extract the PCGs from the each mitogenome of three *Artocarpus* species. Calculate relative synonymous codon usage (RSCU), GC content, and effective number of codons (ENC) were analyzed using CodonW v1.4.4 software combined with online tool CUSP (Sharp and Li, 1986; Burland, 2000). R package ‘ggplot2’ was further employed to generate ENC-plot with GC3 on the x-axis and ENC on the y-axis. The theoretical ENC values were calculated using formula (1) (Romero et al., 2000). And a standard curve was constructed by plotting GC3 values on the x-axis and theoretical ENC values on the y-axis. The ratio of actual ENC to theoretical ENC was subsequently calculated using Equations 1, 2 followed by the generation of a frequency distribution table for the ENC ratios. The neutral plot was created with GC12 on the y-axis and GC3 on the x-axis, with the y=x line as a reference.

Formula:

(1) $$\text{ENC}=\;2+\text{GC}3\;+\;29/\;[\text{GC}3^{2}+(1-\text{GC}3{)}^{2}]$$

(2) ENC ratio=(theoritical ENC −actual ENC)/actual ENC

Data visualization was performed using R v3.6.0. RNA sequencing data from *A. integer*, *A. heterophyllus*, and *A. heterophyllus*(R) (SRR31809564, SRR31809563, SRR31809562) were aligned to CDS sequences using Bowtie2 v2.4.1 and sorted with samtools v1.9 (Li et al., 2009). Then, the bcftools software (v 1.10.2) was used to identify the SNP sites between the sequencing data and the genome, and these sites were regarded as potential RNA editing sites (Narasimhan et al., 2016).

### 2.4 Ka/Ks analyses

Species were paired for comparative analysis, and homologous gene pairs were identified. Multiple sequence alignments of the homologous gene pairs were performed using MAFFT v7.427 (Katoh and Standley, 2013), incorporating the following species: *A. heterophyllus* (PQ839731), *A. heterophyllus*(R) (PQ839730), *A. integer* (OP032238), *Cannabis sativa* (KU310670), *Crataegus pinnatifida* (OR448911), *F. carica* (OQ629317), *Hemiptelea davidii* (MN061667), *Hippophae tibetana* (PP712939), *M. notabilis* (MK301435), *Rosa chinensis* (OP177682), *R. hybrid* (OQ628291), *R. laevigata* (PQ149012), *Ziziphus jujuba* (PP035764). Following alignment, non-synonymous (Ka) and synonymous (Ks) substitution rates for each gene pair were calculated using KaKs Calculator v2.0 with the MLWL method (Zhang et al., 2006). Subsequently, Ka/Ks ratios were computed and visualized as box plots and bar charts using the ggplot2 package in R (Ito and Murphy, 2013).

### 2.5 Sequence collinearity analyses

Homologous sequences between the three *Artocarpus* species and other 10 selected species—including *C*. *pinnatifida*, *C*. *sativa*, *R*. *chinensis*, *R. hybrid*, *R. laevigata*, *F*. *carica*, *M*. *notabilis*, *H*. *davidii*, *H*. *tibetana*, and *Z*. *jujuba*—were identified using BLASTN v2.9.0+ (word size=7, E-value threshold=1e-5). TBtools v2.119 was then used for visualization.

### 2.6 Phylogenetic tree analyses

To determine the phylogenetic position of 88 species in Rosales were downloaded from the NCBI database to construct a phylogenetic tree. Nine homologous single copy genes (*atp1*, *atp9*, *ccmB*, *ccmFc*, *ccmFn*, *cox2*, *matR*, *mttB* and *nad6*) were extracted using Perl scripts from each mt genomes and further MAFFT software (v7.429) was used to multiple sequence alignment (Katoh and Standley, 2013). Subsequently, ambiguously aligned regions were refined using Gblocks 0.91b (Talavera and Castresana, 2007) to improve alignment accuracy and remove poorly conserved positions, and the sequences were pieced together to construct a system evolution tree. Finally, the phylogenetic tree was then constructed using IQ-TREE-1.6.12 by applying the maximum likelihood method (Darriba et al., 2012). The detection of base substitution models uses the built-in model finder of IQ-TREE, and the optimal nucleic acid substitution model is: TIM+F+R3. Setting the bootstrap value at 1000. The resulting tree diagram was visualized using Figtree v. 1.4.4 (http://tree.bio.ed.ac.uk/software/figtree/) (Price et al., 2010), and refined with Adobe Illustrator CS6.

## 3 Results

### 3.1 Assembly, and annotation of mitochondrial genome of three *Artocarpus* species

**Figure 1:**
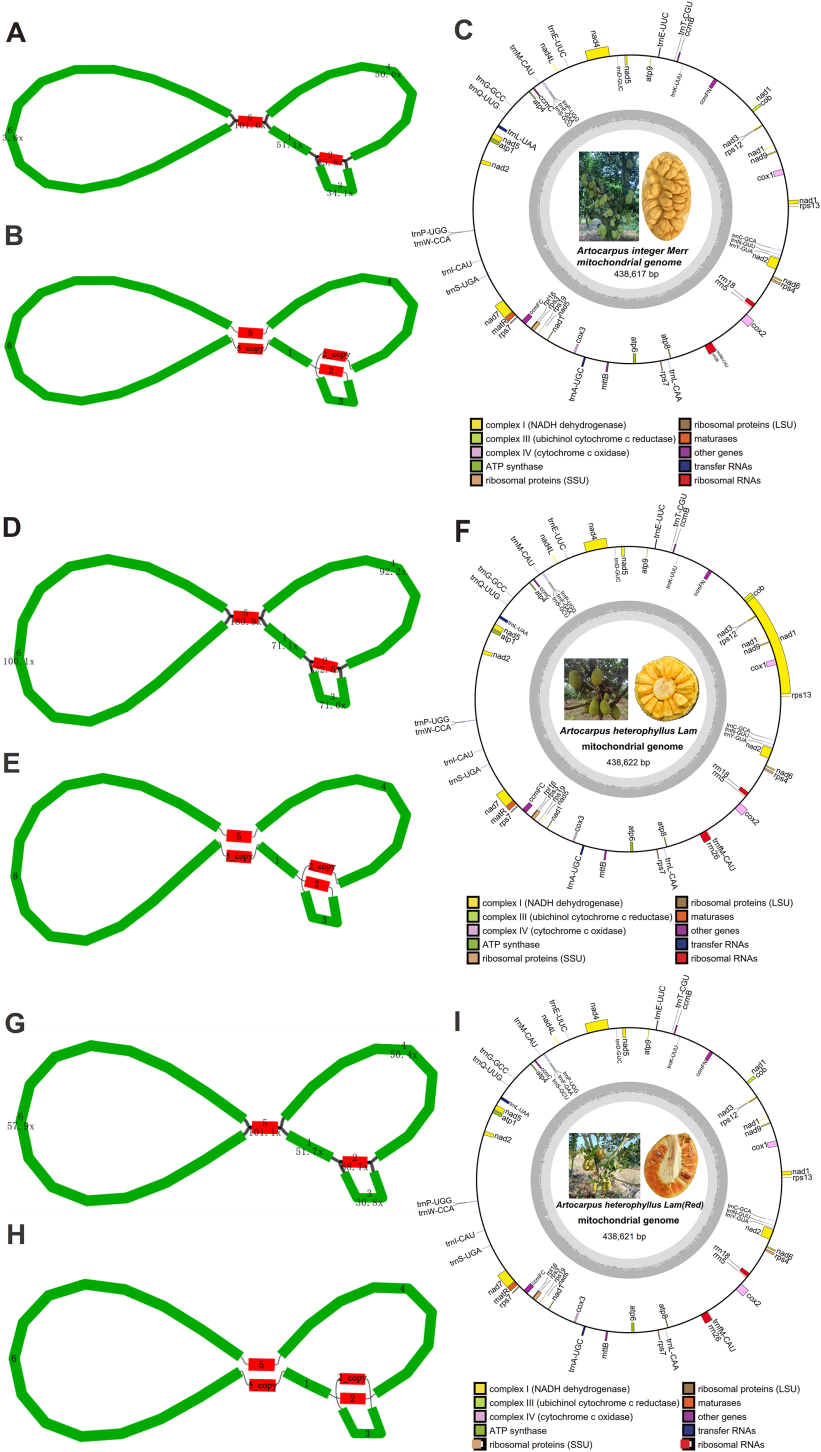
Circular maps of three mitogenomes in *A integer***(A)**, *A. eterophyllus***(C)**, and *A*. *heterophyllus*(R) **(E)**. Genes in the outermost ring are color-coded according to their functional groups. The assembly graph of *A integer***(B)**, *A. heterophyllus***(D)**, and *A. heterophyllus* (R) **(F)** mitogenomes displayed in Bandage. The mt contigs are depicted using segments of various colors.

The HiFi sequencing generated a total of 5.5 Gb, 5.0 Gb, and 5.6 Gb in three *Artocarpus* species, including *A. integer*, *A. heterophyllus*(R), and *A. heterophyllus*, respectively, with a mean read length of 16,420, 16,036, 17,873, respectively. The basic mitochondrial genome conformations of *Artocarpus* species exhibited multi-branched conformation composed of six cotings (**Figures 1A, D, G**), and the length and depth of cotings were showed in **Table 1**. After de-catenation, three mt genome were composed of one contiguous sequence (**Figures 1B, E, H**), with lengths of 438,617 bp, 438,621 bp, and 438,622 bp, and GC contents of 44.93%, 44.93%, and 44.93%, respectively. For convenience of description and subsequent analysis, we organized the circular molecule in the order contig 1–2–3–2_copy–4–5–6–5_copy–1. Overall, three *Artocarpus* species is a putative one-ring DNA (**Figures 1C, F, I**). Three mitochondrial genome sequences deposited in the GenBank database under the accession numbers PQ839731, PQ839730, and OP032238. The master.gfa file generated by PMAT was provided in the **Supplementary Materials**.

**Table 1:** Statistics of the mitochondrial DNA of three *Artocarpus* species.

Type	*A. integer*	*A. heterophyllus* (R)	*A. heterophyllus*
Total Length	438,617 bp	438,621 bp	438,622 bp
GC%	44.93%	44.93%	44.93%
coting1	234,612 bp (53.6 X)	234,613 bp (100.1 X)	234,612 bp (57.9 X)
coting2	8,493 bp (101.0 X)	8,494 bp (180.9 X)	8,494 bp (101.1 X)
coting3	17,579 bp (51.1 X)	17,579 bp (71.1 X)	17,579 bp (51.7 X)
coting4	8,627 bp (85.8 X)	8,628 bp (122.6 X)	8,628 bp (68.7 X)
coting5	34,142 bp (34.1 X)	34,142 bp (71.0 X)	34,142 bp (30.8 X)
coting6	118,044 bp (50.0 X)	118,044 bp (92.2 X)	118,044 bp (50.4 X)

Further annotation result showed that three species showed high similar mitochondrial genome component, they all contained 32 protein-coding genes (PCGs), 22 tRNA genes, and 3 rRNA genes. The PCGs include 24 core genes and 8 non-core genes. And the 24 core genes consist of 5 ATP synthase genes (*atp1*, *atp4*, *atp6*, *atp8*, and *atp9*), 9 NADH dehydrogenase genes (*nad1*–*nad9*), 4 ubiquinol cytochrome c reductase genes (*ccmB*, *ccmC*, *ccmFC*, and *ccmFN*), 3 cytochrome c oxidase genes (*cox1*–*cox3*), 1 translocon protein gene (*mttB*), 1 maturation enzyme gene (*matR*), and 1 cytochrome c biogenesis gene (*cob*). Additionally, the 8 non-core genes include 1 large ribosomal subunit gene (*rpl16*) and 7 small ribosomal subunit genes (*rps3*, *rps4*, *rps7(x2)*, *rps12*, *rps13*, and *rps19*). Furthermore, 23 introns were identified across 9 PCGs: four in each of *nad1*, *nad2*, *nad5*, and *nad7*; three in *nad4*; and one each in *atp6*, *ccmFC*, *cox1*, and *cox2*.

### 3.2 Codon usage analysis

**Figure 2:**
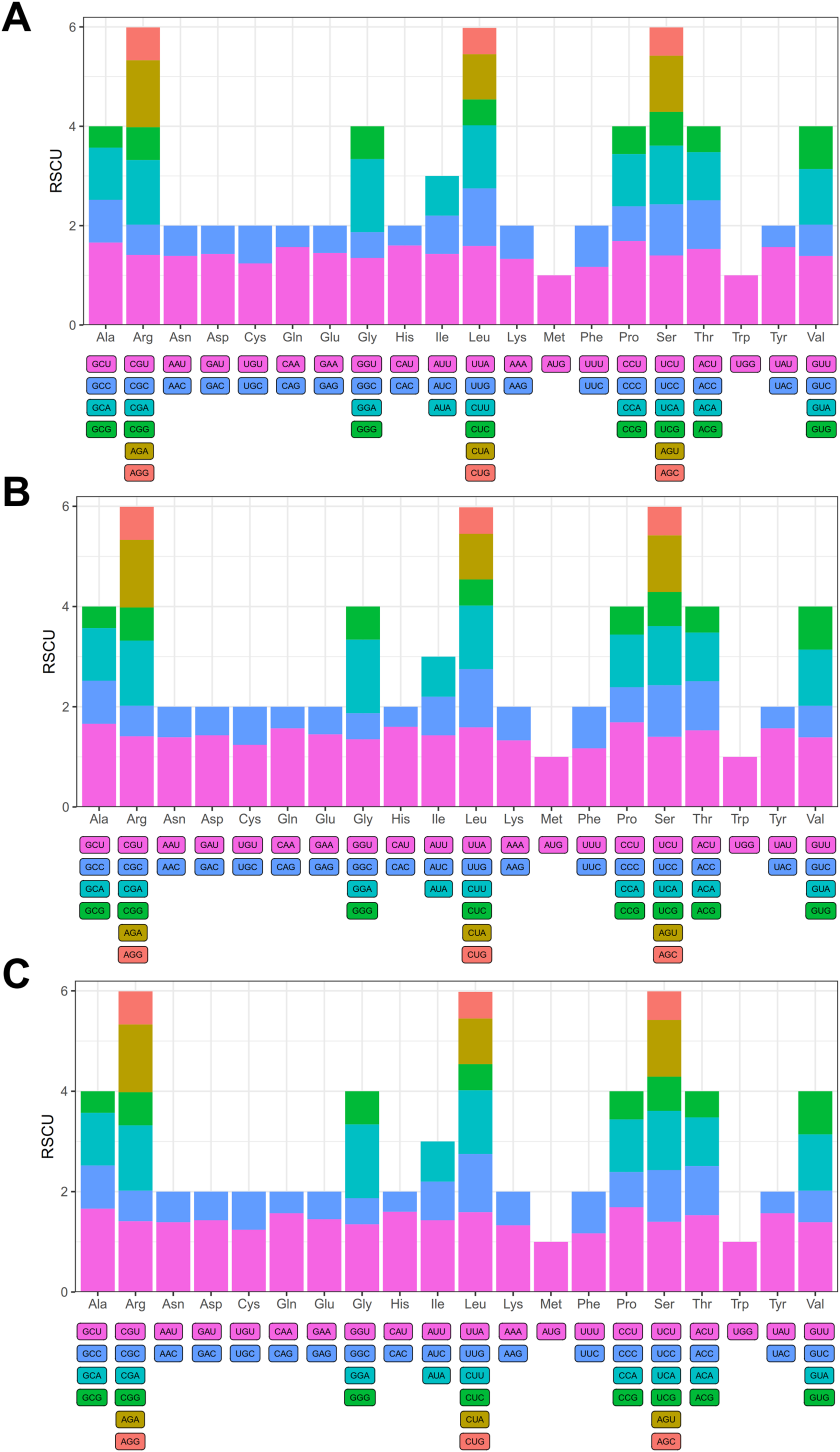
Relative synonymous codon usage (RSCU) in the three mitochondrial genomes including *A. integer***(A)**, *A. heterophyllus***(B)**, *A. heterophyllus*(R) **(C)**. The X-axis shows amino acids, and the Y-axis shows RSCU values for codons.

Three species exhibit identical RSCU (Relative Synonymous Codon Usage) values (**Figure 2**), Most codons demonstrate usage biases in mitochondrial PCGs, with the exception of start codon (AUG) and tryptophan (UGG), both of which have an RSCU value of 1. Among the codons exhibiting usage biases 4,978 codons have an RSCU value greater than 1, and their third position is predominantly A or U, with the exception of UCC and UUG. Furthermore, 2,559 codons possess an RSCU value less than 1, and their third position is primarily G or C (**Supplementary Table S1**). Notably, proline shows a strong preference for the CCU codon in three *Artocarpus* mitochondrial genomes, with its RSCU value reaching as high as 1.69 in mitochondrial PCGs.

**Figure 3:**
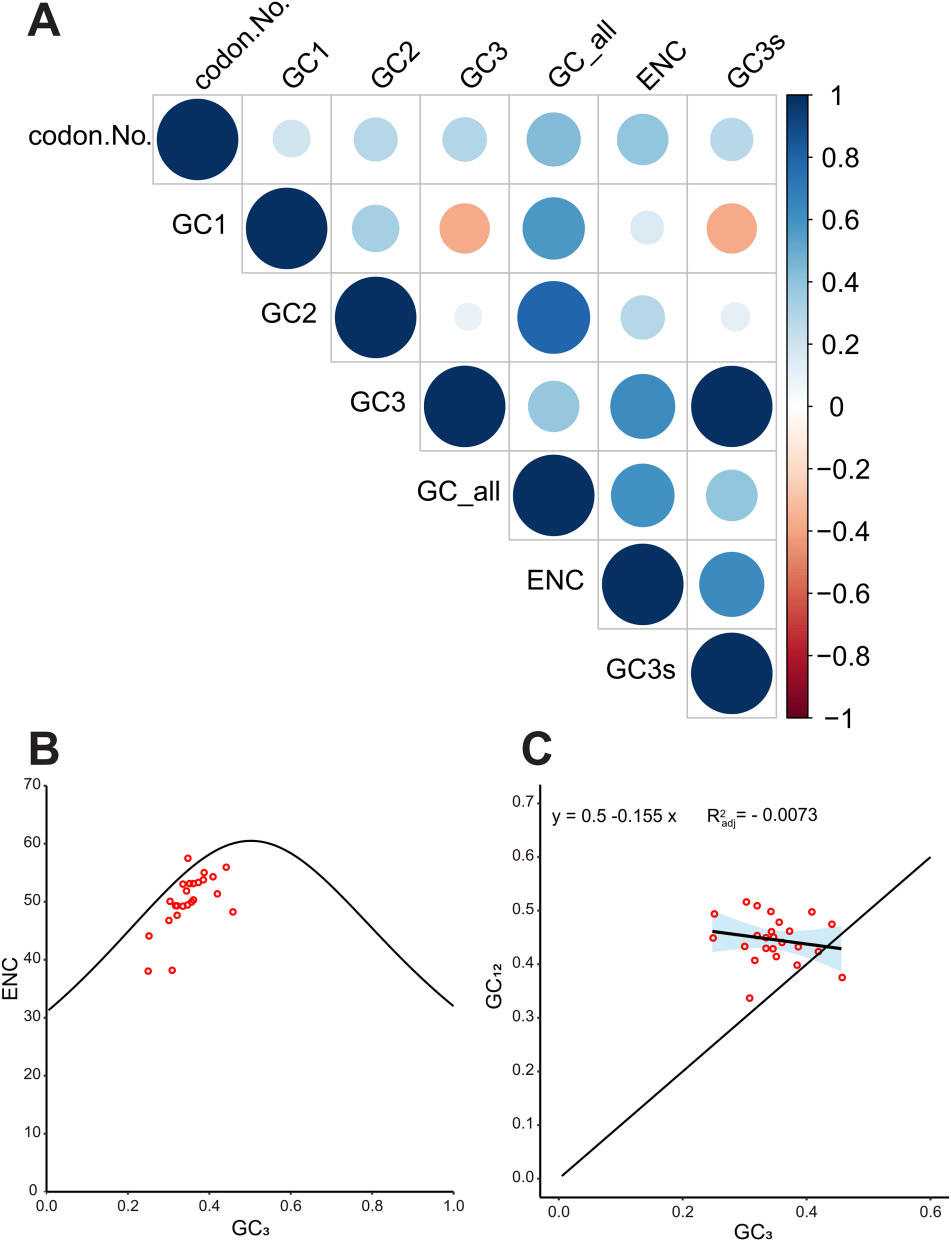
Analysis of correlation of GC content and ENC value **(A)**, ENC plot **(B)** and neutrality plot **(C)** in the *A. integer* mitochondrial DNA.

We further analyzed the base composition of codons. Results showed that the GC contents at positions GC1, GC2, GC3, and GCall were 35.62%~57.84%, 35.44% ~49.44%, 30.53% ~58.54%, 32.67% ~46.74%, and their average values were as follows: GC1(47.50%)> GC2 (41.67%) > GCall (41.30%) > GC3 (34.73%). This indicates that C/G bases prefer to occur in the middle position of each codon in three *Artocarpus* species mt genome. In addition, we also examined the correlation between GC content and the ENC value in three *Artocarpus* species mt genome (**Figure 3A**, **Supplementary Figures S1**). The results showed that GC1 exhibits a positive correlation with GC2, a negative correlation with GC3, and significant correlations with both GCall and ENC (P< 0.001). GC2 was positively correlated with GC3 and ENC, and exhibited a high correlation with GCall (P< 0.001). GC3 demonstrated a positive correlation with GCall and a strong correlation with ENC (P< 0.001). Lastly, a high degree of correlation was observed between GCall and ENC (P< 0.001).

We further generated an ENC-plot graph to illustrate the relationship between GC3 and ENC in three *Artocarpus* species mt genome (**Figure 3B**, **Supplementary Figures S1**). The result indicated that gene distribution is relatively broad, and most genes were distributed below the standard curve and significantly distant from it, while only a few genes were located near the upper part of the standard curve, this observation suggests that codon usage bias in most genes was primarily shaped by natural selection rather than mutational pressure.

Furthermore, we constructed a neutrality plot (**Figure 3C**, **Supplementary Figures S1**), a low regression line slope (0.155) indicates a weak correlation between GC3 and GC12, further suggesting that base mutations play a limited role in shaping codon usage bias in the *Artocarpus* species genome.

### 3.3 Repeat sequence analysis

**Figure 4:**
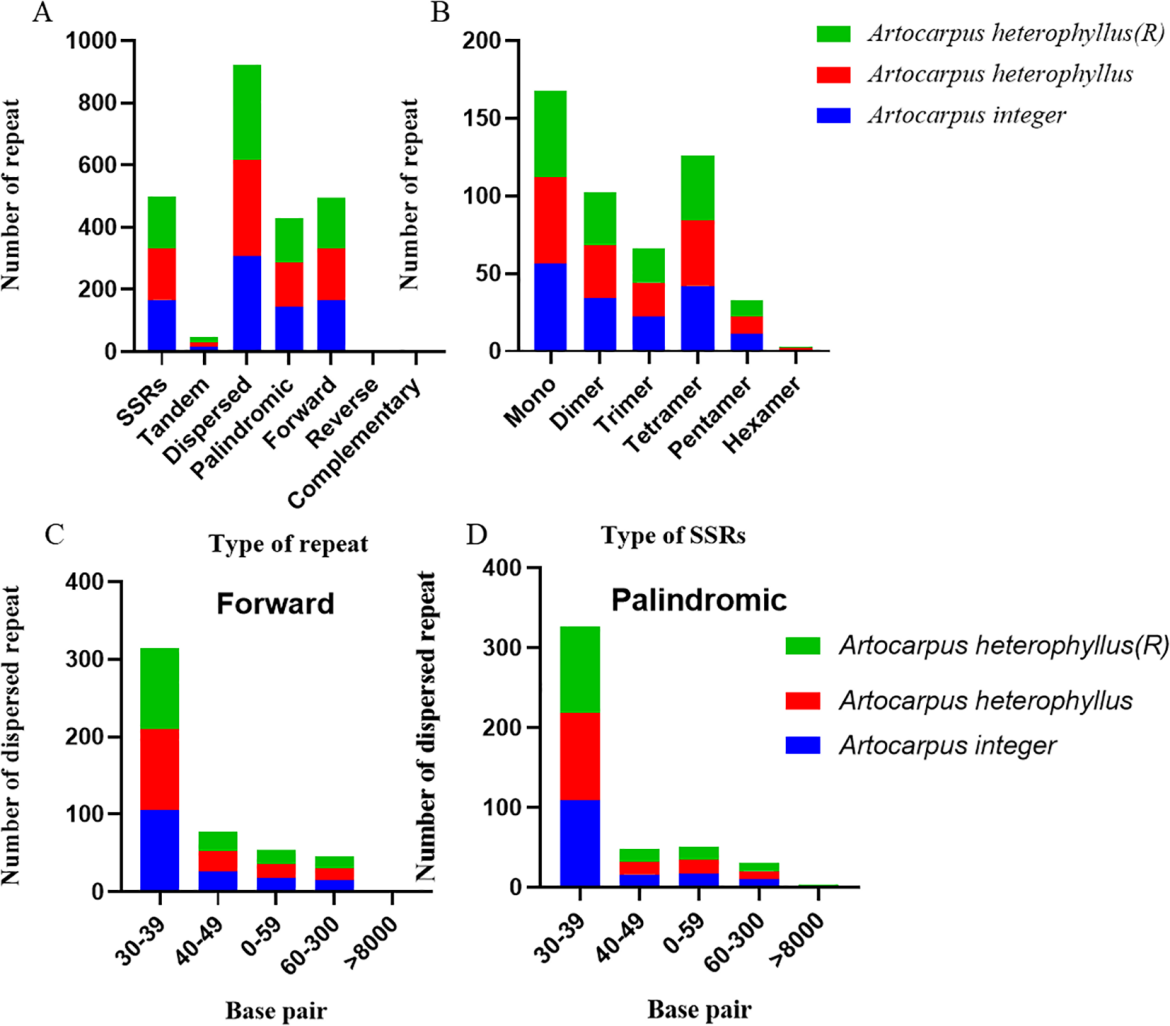
The repeat of three *Artocarpus* species mitochondrial genome: Type and number of repeat sequences in mitogenome **(A)**. Type and number of SSRs in mitogenome **(B)**. Size and number of forward repeats in mitogenome **(C)**. Size and number of palindromic repeats in mitogenome **(D)**.

The mitochondrial DNA of *Artocarpus* species contains three types of repeated sequences, including simple sequence repeats (SSRs), tandem repeats, and dispersed repeats (**Figure 4A**). A total of 166 SSRs were identified, with mononucleotide repeats being the most prevalent, accounting for 33.73%. This was followed by trinucleotide repeats (25.30%), dinucleotide repeats (20.48%), tetranucleotide repeats (13.25%), pentanucleotide repeats (6.63%), and hexanucleotide repeats (0.60%) (**Figure 4B**). Among SSRs the mononucleotide repeat sequence A/T was identified as the most abundant type, totaling 51 occurrences and accounting for 91.07%. The dinucleotide repeat sequence AT/AT, was the second most abundant, with a total of 18 occurrences, Additionally, 17 tandem repeat sequences were detected, exhibiting a sequence identity of ≥75% and lengths ranging from 12 to 42 bp (**Figure 4A**). Furthermore, a total of 308 pairs of dispersed repeats were identified, each with a length of at least 30 bp, including 165 pairs of forward repeats and 153 pairs of palindromic repeats (**Figures 4C, D**). Notably, no reverse or complementary repeats were found. Moreover, all three species contained two repetitive sequences larger than 8,000 bp (**Figure 4D**).

**Figure 5:**
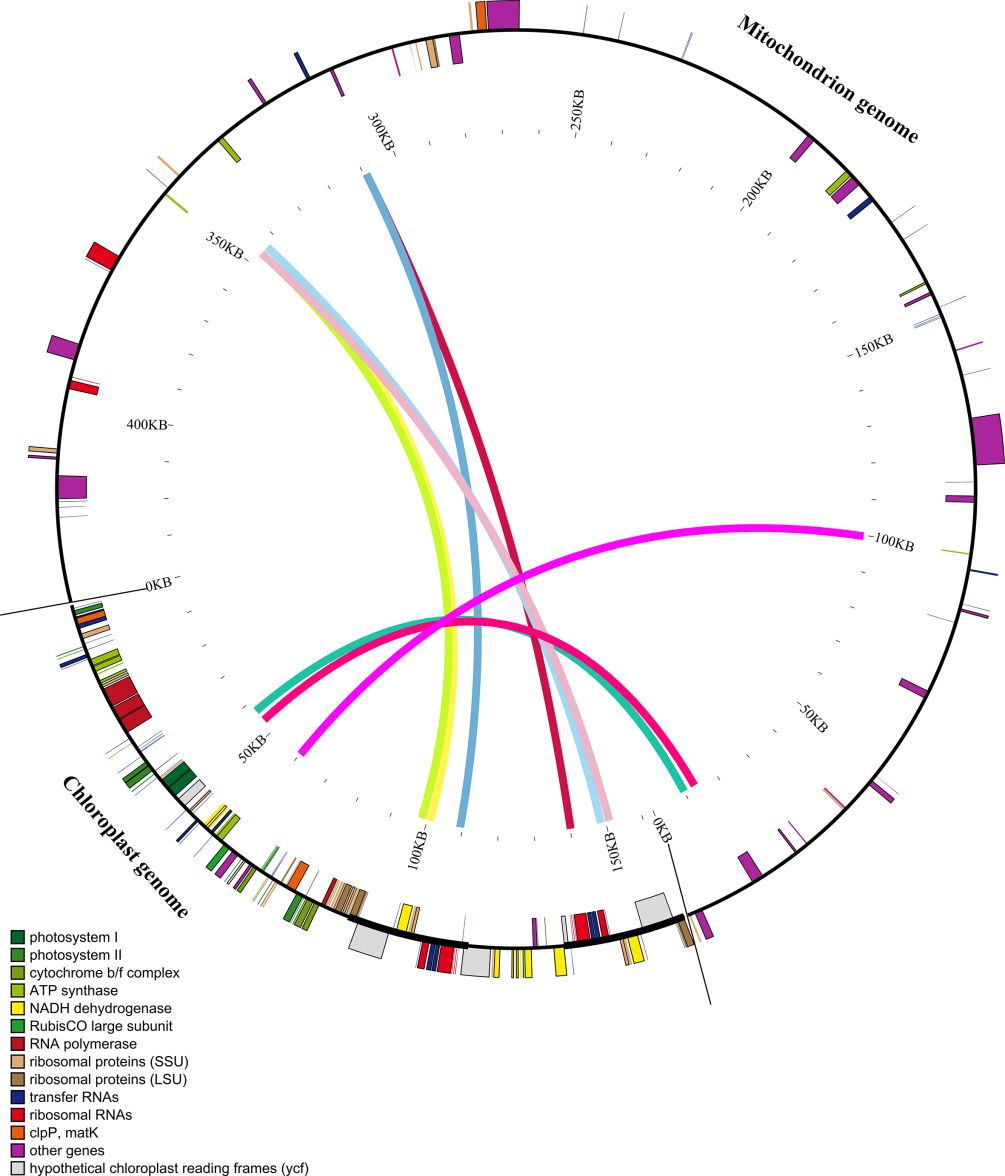
Locations of the transferred fragments between mitochondrial and chloroplast DNA of *A.integer* (PQ839729.1, PQ835411.1). The genomic segments corresponding to the lines between the arcs in the figure are homologous fragments longer than 1,000 bp between the chloroplast and the mitochondrion.

Based on the sequence similarity in nucleotide sequences between mitochondrial and chloroplast DNA, we identified nine homologous fragments between the two organelle genomes (**Figure 5**). The total length of these fragments is 24,398 base pairs, with the longest fragment reaching 5,149 base pairs. Further annotation of these homologous sequences revealed six complete genes, which include four protein-coding genes (*psaB*, *psaA*, *ndhB*, *rps*7) and two tRNA genes (*trnL-CAA*, *trnA-UGC*). Additionally, only fragments of the genes *ycf3*, *rbcL*, *ycf2*, *rps12*, *rrn23*, and *trnI-GAU* were captured; the block boundaries fall within these genes, indicating that the transfers comprise gene pieces rather than full-length loci (**Table 2**).

**Table 2:** Genes identified between cp and mt genomes.

Block	Source	Start	End	Length	Gene
1	cp	40310	45495	5186	*psaB*, *psaA*
	mt	7438	12586	5149	–
2	cp	45667	46787	1121	ycf3*
	mt	12770	13870	1101	–
3	cp	59642	60645	1004	rbcL*
	mt	99242	100237	996	–
4	cp	97842	98963	1122	ycf2*, *trnL-CAA*
	mt	345390	346511	1122	*trnL-CAA*
5	cp	98988	102624	3637	*ndhB*, *rps7*, rps12*
	mt	341758	345375	3618	*rps7*
6	cp	107434	111279	3846	trnI-GAU*, *trnA-UGC*, rrn23*
	mt	307359	311194	3836	*trnA-UGC*
7	cp	138190	142035	3846	rrn23*, *trnA-UGC*, trnI-GAU*
	mt	307359	311194	3836	*trnA-UGC*
8	cp	146845	150481	3637	rps12*, *rps7*, *ndhB*
	mt	341758	345375	3618	*rps7*
9	cp	150506	151627	1122	*trnL-CAA*, ycf2*
	mt	345390	346511	1122	*trnL-CAA*

### 3.4 Conserved block analysis

**Figure 6:**
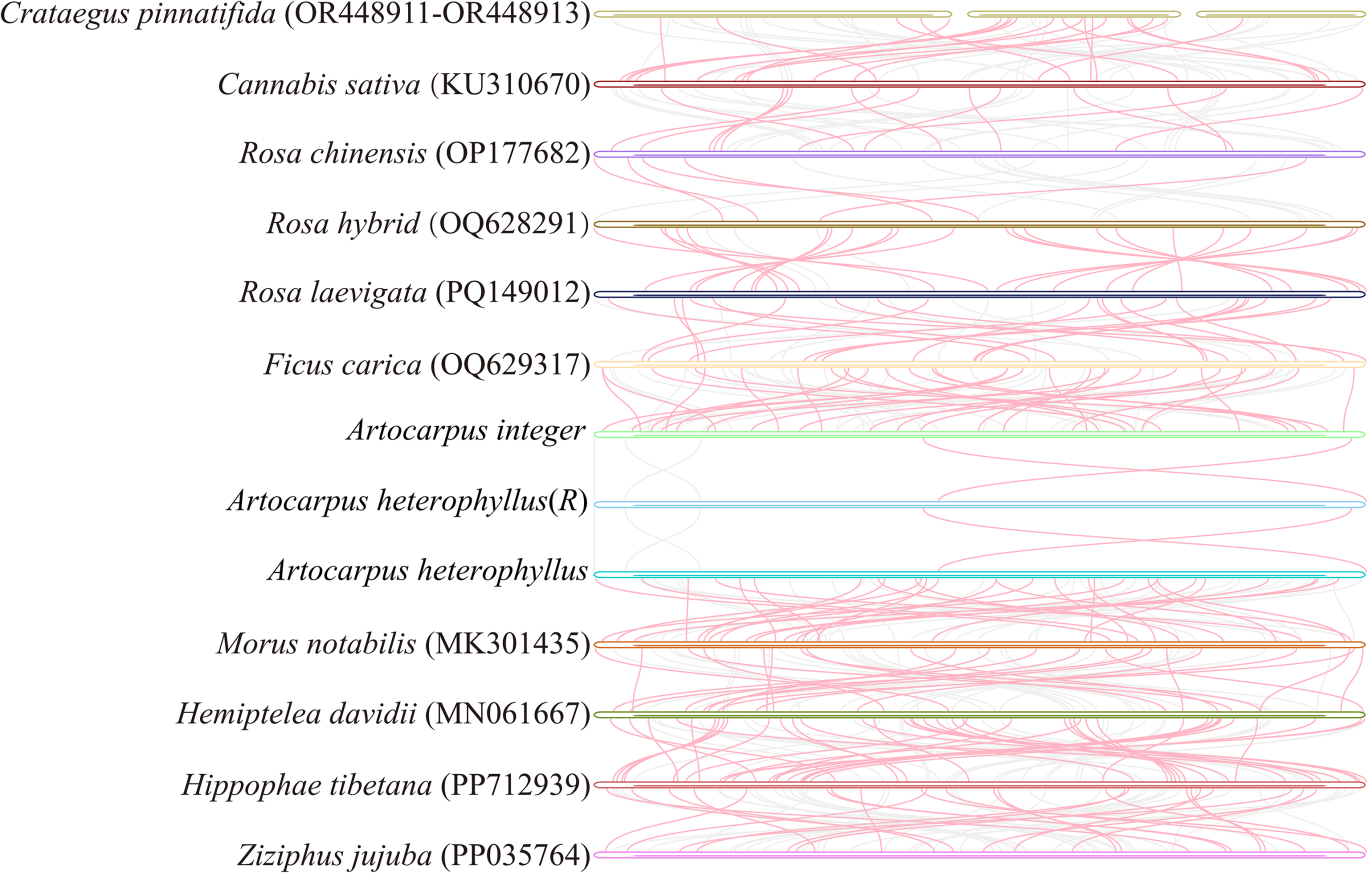
Collinear analysis between three *Artocarpus* species and other ten *Rosales* species. The curved areas connected by lines in the figure represent regions of high similarity, with red arcs representing reverse sequences and gray areas representing forward sequences.

A comparative analysis of collinearity was conducted among three *Artocarpus* species and ten additional species within the order Rosales (**Figure 6**), including *C. pinnatifida*, C. *sativa*, *R. chinensis*, *R. hybrid*, *R. laevigata*, *F. carica*, *A. integer*, *A. heterophyllus*(R), *A. heterophyllus*, *M. notabilis*, *H. davidii*, and *H. tibetana*. The results indicated that a substantial number of homologous syntenic fragments were identified across the 13 mitochondrial genomes within the order Rosales, including numerous syntenic blocks exceeding 1000 bp in length (**Figure 6**, **Supplementary Table S2**). Notably, the three *Artocarpus* species share extensive homologous segments, and the total length of their collinear blocks is the longest among all species examined, indicating three species exhibit a high degree of conservation in their mitochondrial genomes. In addition, the inconsistent arrangement of syntenic blocks among these mitochondrial genomes indicated extensive genomic rearrangements, reflecting a highly divergent and structurally non-conserved mitochondrial genome architecture.

### 3.5 Ka/Ks analysis

**Figure 7:**
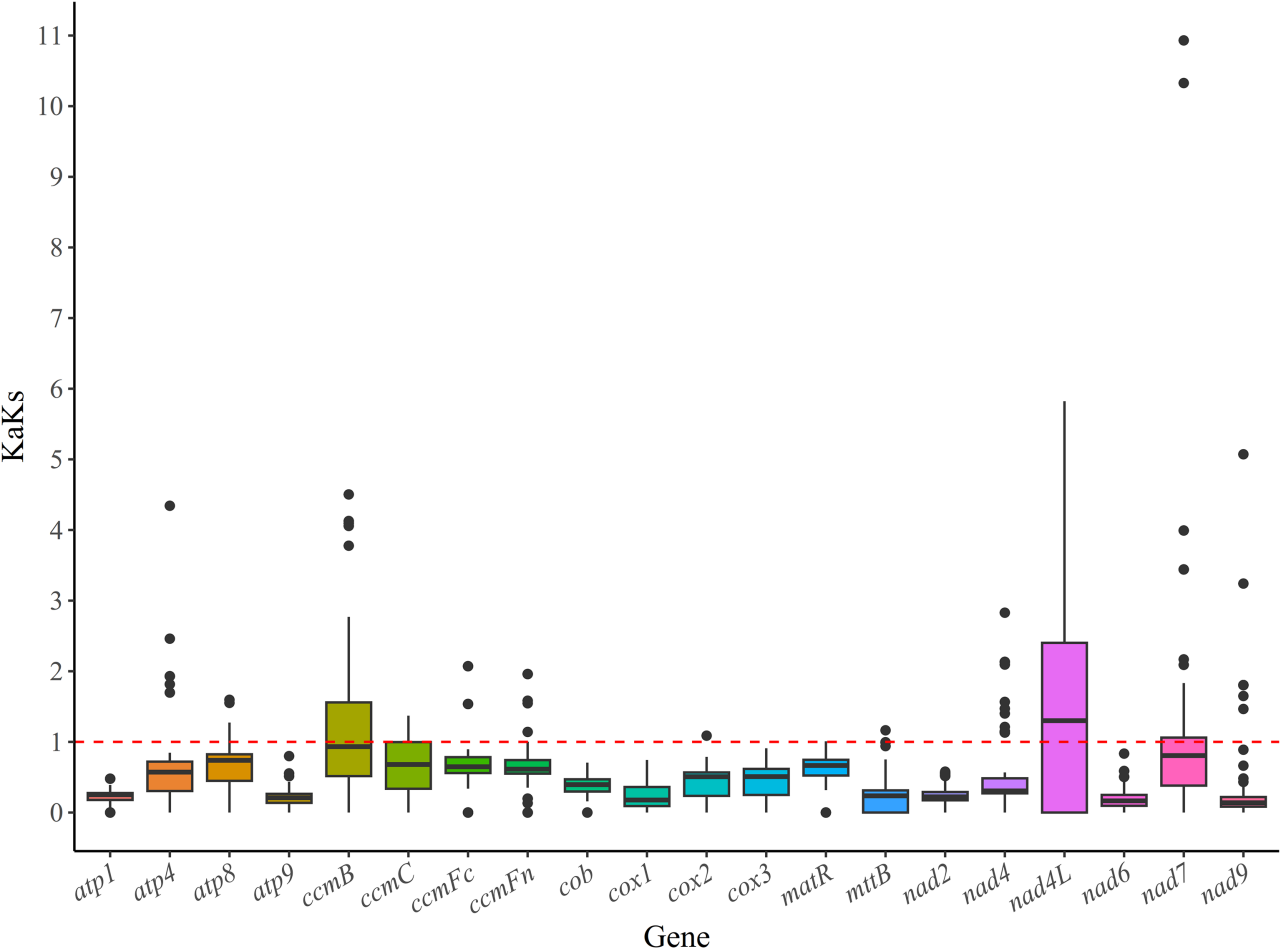
Analysis of the Ka/Ks ratios for 18 PCGs between three *Artocarpus* species and ten additional *Rosales* species.

We examined the Ka/Ks ratios of 18 PCGs across three *Artocarpus* species, contrasting with 12 other species from the *Rosales* order (**Figure 7**). The average Ka/Ks ratios for 14 PCGs (*atp1, atp4, atp8, atp9, ccmFn, cob, cox1, cox2, cox3, matR, mttB, nad6, nad7 and nad9*) were found to be less than 1, with *atp9* (Ka/Ks = 0.19) and *nad6* (Ka/Ks = 0.23) representing the lowest values. This indicates that 14 PCGs have undergone purifying selection during evolution and possess relatively stable protein functions. In contrast, the average Ka/Ks ratios for *ccmB*, *ccmC*, *ccmFc*, and *nad4L* were exceeded 1, *nad4L* (Ka/Ks = 1.53) and *ccmB* (Ka/Ks = 1.37) were strongly and positively selected. The KaKs analysis results indicated that the majority of PCGs have undergone purifying selection. .

### 3.6 Phylogenetic analysis

**Figure 8:**
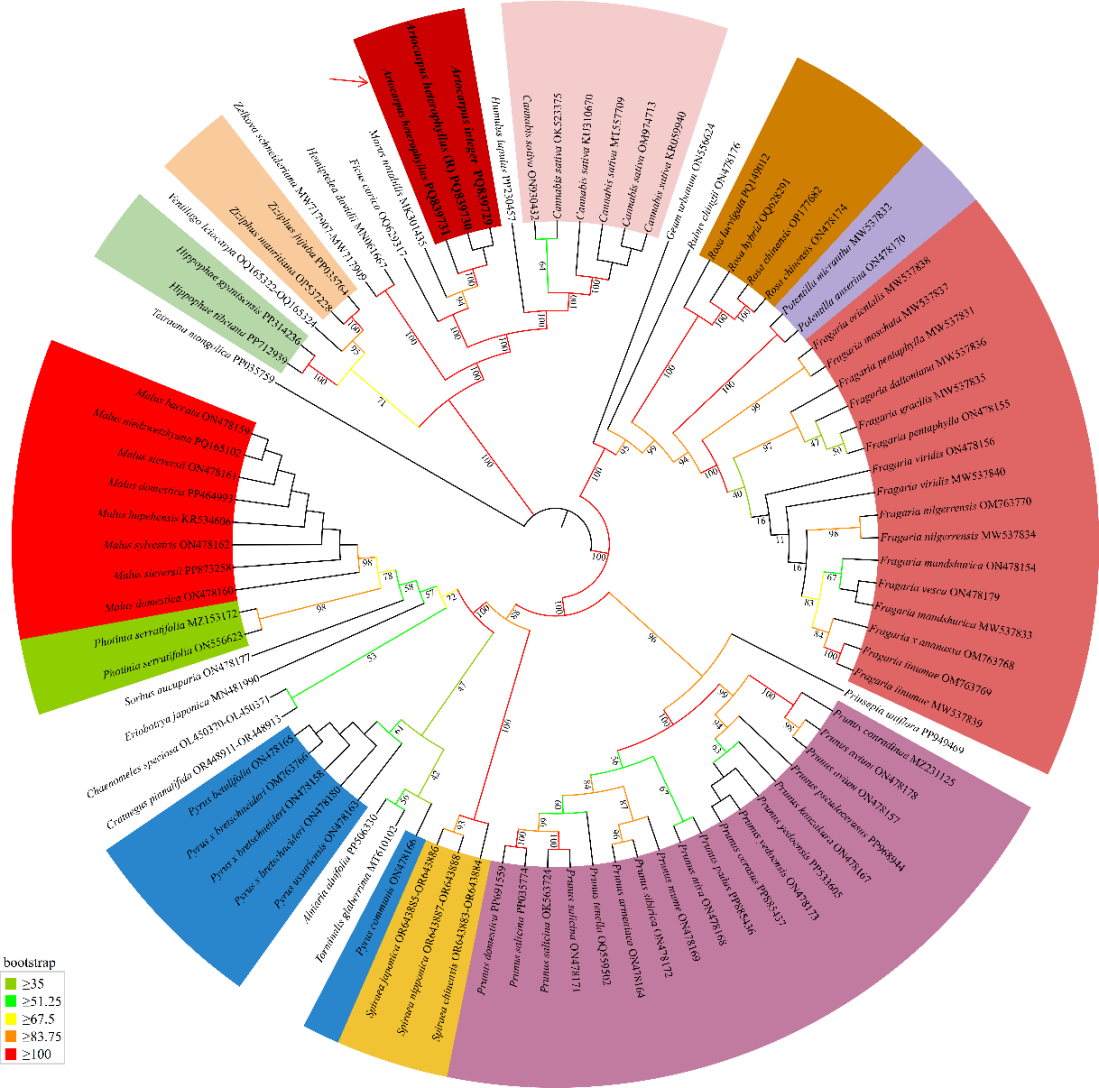
Maximum likelihood tree based on the 88 species.

To ascertain the phylogenetic status of three *Artocarpus* species, we constructed a maximum likelihood tree utilizing DNA sequences from nine single-copy orthologous genes across 88 species, including *ccmB*, *ccmFc*, *ccmFn*, *cox2*, *matR*, *mttB*, *atp1*, *atp9*, and *nad6*, with the *Tetraena mongolica* serving as the outgroup, and different leaf colors represent different genera (**Figure 8**). The phylogenetic analysis indicates that the three *Artocarpus* species exhibit a high degree of closure and are closely related to *M. notabilis* and *F. carica*. In addition, the Moraceae family is phylogenetically closely related to the families Cannabaceae and Ulmaceae.

### 3.7 RNA editing analysis

**Figure 9:**
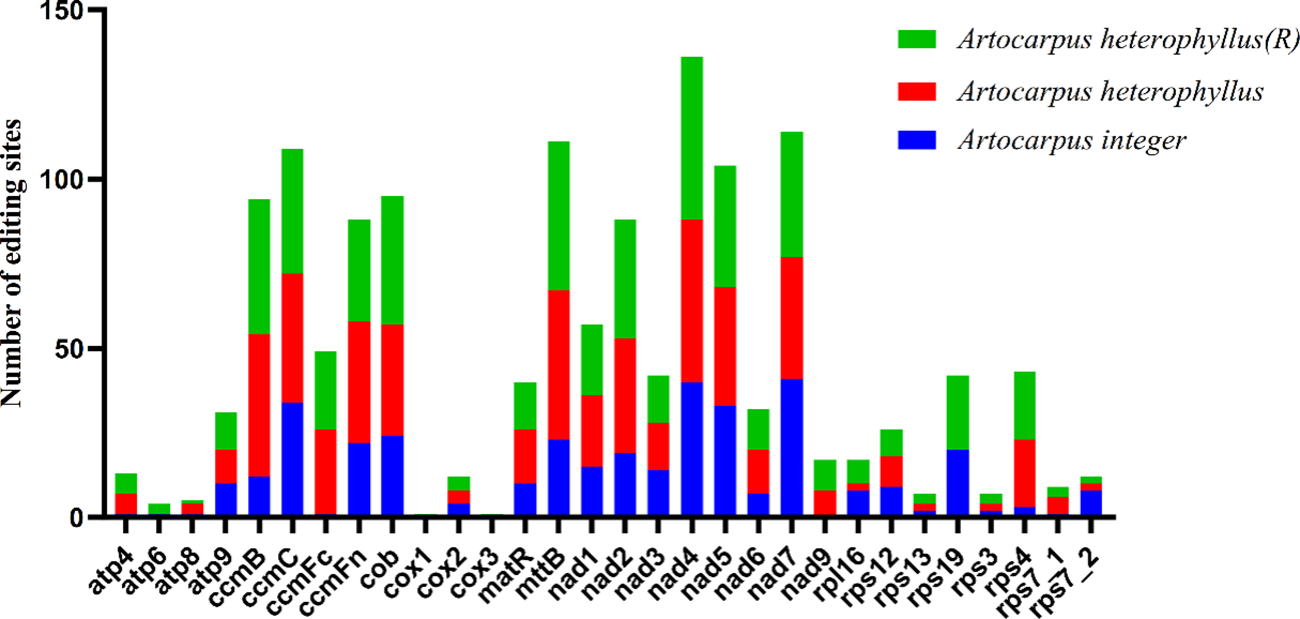
Number of RNA editing sites in 30 shared PCGs in the mitogenomes of three *Artocarpus* species.

RNA editing sites were predicted using RNA-Seq data from the PCGs in the mitogenomes of three *Artocarpus* species. The results showed that a total of 365 RNA editing sites were predicted in *A. integer*, which is fewer than the 533 sites in *A. heterophyllus* (R) and the 508 sites in *A. heterophyllus* (**Table 3**). And 8 PCGs exhibited significant differences (**Figure 9**, **Table 3**), including *atp*4, *atp*6, *atp*8, *cox*1, *cox*3, *rps*4, *rps*19 and *rps*7-2. Notably, the *cox*1 and *cox*3 genes were exclusively predicted in *A. heterophyllus* (R).

**Table 3:** Statistics of RNA editing number of three Artocarpus species.

Type	*A. integer*	*A. heterophyllus* (R)	*A. heterophyllus*
Total edits	365	533	508
Total PCGs edits	291	482	485
Proportion C-to-T(U)	0.7973	0.9043	0.9547
Total U(T)-to-C edits	11	5	1
Highest editing gene (edits)	nad7 (41)	nad4 (48)	nad4 (48)
Second edits gene (edits)	*nad4* (40), *ccmC* (34)	*mttB* (44), *ccmB* (40)	*mttB* (44), *ccmB*(42)
Less edits gene (edits)	atp4 (1), atp6 (1), atp8 (1), ccmFc (1), rps7_1(1)	atp8 (1), cox1 (1) cox3 (1)	rpl16 (2), rps3 (2), rps7_2 (2)
Proportion of hydrophilic to hydrophobic (%)	34.52	39.40	40.55

The reverse U(T)-to-C editing sites were also predicted in three *Artocarpus* species, revealing significant differences among them. Notably, *A. integer* exhibited the highest RNA editing frequency with 11 edits, followed by *A. heterophyllus* (R) with 5 edits, and *A. heterophyllus* with only 1 edit. Detailed statistics of U(T)-to-C RNA editing are presented in **Supplementary Tables S3**-**Supplementary Tables S5**. The results indicate that reverse U(T)-to-C editing of the *cob* gene was detected in all three species. However, the *rpl*16 and *rps*19 genes were not detected in *A. heterophyllus*, while the *ccmFn*, *nad*7, *matR* and *rps*7–2 genes were exclusively detected in *A. integer.*

## 4 Discussion

Species within the *Artocarpus* genus, including *A. integer*, *A. heterophyllus*, and *A. heterophyllus* (R), possess significant edible and medicinal value (Gupta et al., 2023). These species are extensively cultivated on Hainan Island, contributing substantially to the local economy. The assembly and analysis of plastomes are essential for advancing plant breeding programs and other agronomic purposes (Kan et al., 2024). Plant mitochondrial DNA is crucial as organelle DNA exhibits a high degree of conservation and evolves at a distinct rate compared to nuclear genes, rendering it an invaluable molecular marker for studying evolution and molecular ecology (Zubaer et al., 2018). In the NCBI database, a limited number of mitochondrial DNA sequence have been documented in the *Moraceae* family (Liangliang, 2022; Wei et al., 2023). It is noteworthy that no reports currently exist on the mitochondrial genome of *Artocarpus* plants. Which has hindered our understanding of the mitochondrial genome structure with the *Artocarpus* genus and impeded research into its evolutionary relationships and molecular breeding.

Compared to animal mitochondrial DNA, plant mitochondrial DNA exhibit greater variability and demonstrates rich diversity in terms of size (ranging from 200 kb to 11 Mb), structure, and gene content across different species, and even within the same species (Barr et al., 2005; Davila et al., 2011; Sloan et al., 2012; Skippington et al., 2015). In this study, we successfully assembled the first complete mitochondrial of *Artocarpus* species using high-quality HiFi reads produced by PacBio technology. The three mitochondrial DNA (mtDNA) sequences contained six cotings and showed highly similarity in mitochondrial genomic size, with only minor base pair differences. Furthermore, all sequence exhibited a GC content of 44.9%, which aligned with that of certain species within the *Moraceae* family, such as *F. carica* (45.45%) (Wei et al., 2023); and *M. alba* (45.50%) (Liangliang, 2022). The *Artocarpus* species show notable similarity in mtDNA size and structure, in contrast to the greater variability observed in other closely related genera, such as *Mangifera* and *Crataegus* (Niu et al., 2022; Zhang et al., 2024), suggesting that *Artocarpus* species may exhibit relative conservation in organelle evolution. Additionally, we assembled three complete chloroplast DNA sequences (**Supplementary Figures S5**, **Supplementary Figures S6**), which also displayed high structural similarity (**Supplementary Table S6**). This finding is consistent with previous conclusions regarding the chloroplast genome of *Artocarpus camans*i (Souza et al., 2020). In conclusion, these results indicated that the organelle genomes of *Artocarpus* species remain highly conserved throughout breeding and evolutionary processes.

Repetitive sequences play a crucial role in promoting genetic recombination in the mitochondrial DNA of seed plants, as they can alter genome size and induce structural variations (Mackenzie, 1999; Chen et al., 2017). These sequences serve as valuable genetic markers in population analysis, thereby facilitating research on species evolution (Provan, 1996; Pfeifer et al., 2013; Li et al., 2022). In this study, we found that three species exhibit a high degree of similarity in their repetitive sequences. A total of 166 SSRs were identified in each mitogenome, which exceeds the number of SSRs found in the chloroplast genome (Souza et al., 2020). Furthermore, mononucleotide A/T repeats were the most frequent, consistent with findings in *Punica granatum* (Feng et al., 2023), and comparable to those in *A. camansi* and *A. heterophyllus* of plastid genomes (Souza et al., 2020). The mitochondrial genome of *Artocarpus* contains up to 308 dispersed repeats exceeding 30 base pairs, as well as a large repeat region greater than 8,000 base pairs. Extended repetitive sequences can promote genomic recombination, leading to structural modifications (Gualberto et al., 2014). Furthermore, 17 tandem repeats were identified, which differ from those found in *F*. carica and *M. alba* (Liangliang, 2022; Wei et al., 2023).

Homologous fragment transfer occurs among the nucleus, mitochondria, and chloroplasts. Frequent DNA transfers are estimated to have taken place in the common ancestor of gymnosperms and angiosperms approximately 300 million years ago (Timmis et al., 2004; Wang et al., 2007). Understanding this transfer relationship is essential for explaining the evolution of plant mitogenomes (Van Binh et al., 2020). In this study, the PCGs of *psaA, PsaB, rps7 and ndhB* were identified as completely transferred sequences between mtDNA and cpDNA of three *Artocarpus* species. The *psaA*, *rps7* and *ndhB* genes were also transferred between organelle genome of *Hibiscus cannabinus* (Moghaddam et al., 2023).These genes play a crucial role in chloroplast functionality (Shikanai et al., 1998; Horvath et al., 2000; Grotjohann and Fromme, 2005).Their presence in the mitochondrial genome most likely represents chloroplast-derived insertions rather than functional mitochondrial genes.

Codon usage bias (CUB) is crucial for investigating the origins of species and their genetic differentiation (Athey et al., 2017). An RSCU value greater than 1 indicates a higher frequency of codon usage, while a value less than 1 suggests lower codon usage frequency (Parvathy et al., 2022). In the genomes of the three *Artocarpus* species, 4,978 codons exhibit an RSCU value above 1, with the third codon position predominantly being either A or U, that is a common feature in terrestrial plant mitochondrial DNA (Fang et al., 2022). Both neutrality plot and ENC-plot analyses indicate that the three *Artocarpus* species are influenced by both mutation and natural selection. However, the influence of natural selection is more dominant, similar to the findings in *Angelica biserrata* and *Rhingia Scopoli* (Wang et al., 2024; Zhao et al., 2024).

The Ka/Ks ratio is frequently employed to assess the selective pressure acting on PCGs, which determines the effects of environmental stress on the evolution of the mitochondrial genome (Qiao et al., 2022). Our results indicated that most PCGs have a Ka/Ks ratio of less than 1, suggesting that the majority of protein-coding genes have undergone purifying selection during evolution, leading to relatively stable protein functions (Zeng et al., 2024). Among these, four genes (*ccmB*, *ccmC*, *ccmFc*, and *nad4L*) exhibited positive selection in *Artocarpus* species. *CcmB* has frequently been identified as a positively selected gene in various plants, including *F*. carica, *Ilex metabaptista*, *Astragalus membranaceus*, *Thuja sutchuenensis*, *Calophyllum soulattri* and *Phaseolus vulgaris* (Bi et al., 2020; Cheng et al., 2021; Xia et al., 2023; Zhou et al., 2023; Cadorna et al., 2024; Zhang et al., 2024). Both *ccmB* and *nad4L* also function as positive selection genes in *Hippophae tibetan*a and *Diospyros kaki* (Yang and Duan, 2024; Zeng et al., 2024). *CcmB* gene plays a significant role in helping plants resist stress and adapt to their environment (Xiong et al., 2017; Xu et al., 2023; Dai et al., 2024). Specially, genes *ccmB, ccmC*, and *ccmFc* are components of the mitochondrial cytochrome c maturation (CCM) complex (Brausemann et al., 2021), they plays a significant role in the repeated evolution of various species (Huang et al., 2024). These positive genes may play a crucial role in adaptation of *Artocarpus* species to tropical environments in Hainan province.

The arrangement of homologous regions has been widely utilized to elucidate the phylogenetic relationships among species (Mower et al., 2012; Zhang et al., 2023). In our study, the collinear blocks involving three *Artocarpus* species were observed to be the longest among all identified blocks. This suggests that more closely related species tend to exhibit elongated collinear regions. Concurrently, we noted distinct differences in the arrangement of these collinear blocks between *Artocarpus* and other species. This indicates that the mitochondrial genome of the three *Artocarpus* species have undergone extensive rearrangements compared to their close relatives, showcasing a high degree of structural variability.

In order to establish the phylogenetic position of *Artocarpus* species based on mt genome, we constructed a phylogenetic analysis utilizing 8 shared PCGs from 88 species. Results indicated that the three *Artocarpus* species are closely related, with the closest phylogenetic relationship observed between these species and *M. notabilis* (MK301435). This finding is consistent with previous classification results based on analyses of the ndhF gene, ITS sequences, and chloroplast DNA (Souza et al., 2020). Furthermore, the three Artocarpus species exhibit a close phylogenetic relationship with *F. carica*. However, this result is inconsistent with evolutionary relationship constructed using chloroplast genome (Liu et al., 2018; Souza et al., 2020). The limited availability of mitochondrial DNA sequences within the Moraceae family currently restricts the scope of our study. More mitochondrial DNA sequences from the Moraceae family should be research, particularly for the genus *Artocarpus* species.

Plant organelle gene expression correlates with a variety of post-transcriptional nucleic acid modifications, among which RNA editing is particularly significant (Castandet and Araya, 2011). Ancient RNA editing factors originated early in the evolutionary history of flowering plants, aiding researchers in tracing the evolutionary trajectory of plants (Hein and Knoop, 2018). In this research, three *Artocarpus* species exhibit a high degree of similarity in mitochondrial characteristics, their RNA editing analysis shows distinct differences. Notably, *A. integer* displays a markedly lower volume of RNA edits compared to the other two species. As plant evolution progresses, RNA editing events tend to diminish, with ancestral lineages typically exhibiting high editing rates in seed plants (Chen et al., 2011). Notably, a gradual reduction in cellular RNA editing rates also observed in evolution of angiosperms (Small et al., 2020). Therefore, in evolutionary terms, the lower number of RNA editing sites of *A. integer* may be a more derived position relative to *A. heterophyllus* and *A. heterophyllus* (R). In addition, C-T (U) types of RNA editing sites were dominant in single-base editing in this study, this finding that aligns with results from other plant mitochondrial genomes (Wang et al., 2024). The proportion of C-T (U) types varied among the three *Artocarpus* species, accounting for 79.73% in *A. integer*, 90.43% in *A. heterophyllus* (R), and 95.47% in *A. heterophyllus*. Correspondingly, *A. integer*, which had the fewest total editing sites, also exhibited the lowest proportion of C-to-U edits. In contrast, the number of reverse U(T)-to-C types in *A. integer* were significantly higher than other two *Artocarpus* species. On the other hand, RNA editing also has the capacity to alter amino acids, thereby modifying thus physical and chemical properties (Mower, 2009). Previously research indicated that a higher proportion of hydrophilic amino acids facilitates protein folding, whereas a reduced proportion contributes to enhanced protein stability (Wang et al., 2024). In our study, the majority of amino acids transitioned from hydrophilic to hydrophobic in three *Artocarpus* species similar to the patterns in *F. carica*, and *M. alba* (Liangliang, 2022; Wei et al., 2023). Additionally, *A. integer* exhibits a lower hydrophilic-to-hydrophobic ratio compared to the other two species, suggesting that its protein structure is more evolutionarily stable.

## 5 Conclusion

In this study, we successfully assembled and annotated the complete mitochondrial DNA of three economically significant tropical crops: *A. integer*, *A. heterophyllus*(R), and *A. heterophyllus*. This study represents the first report of the mitochondrial genome for the genus Artocarpus, further conducted a comprehensive investigation of various aspects based on the mitochondrial genome, including analysis of Ka/Ks ratios, repeat sequences, codon usage preference, RNA editing and evolutionary tree. That provide valuable insights for researchers to understand genetic characteristics, molecular differences, and taxonomic categorization of *Artocarpus* species, contribute to elucidating the evolutionary relationships for taxonomic studies within the Moraceae family.

## Data Availability

The datasets presented in this study can be found in online repositories. The names of the repository/repositories and accession number(s) can be found in the article/**Supplementary Material**.
