# Supplementary material for: Characterization and phylogenetic analysis of the first complete mitochondrial genome sequence of three Artocarpus species in Hainan Province
Source: Front Plant Sci. 2025 Dec 19;16:1733932. doi: 10.3389/fpls.2025.1733932 (PMC12757345; doi:10.3389/fpls.2025.1733932)
Supplement: Supplementary file 1 [file DataSheet1.docx]

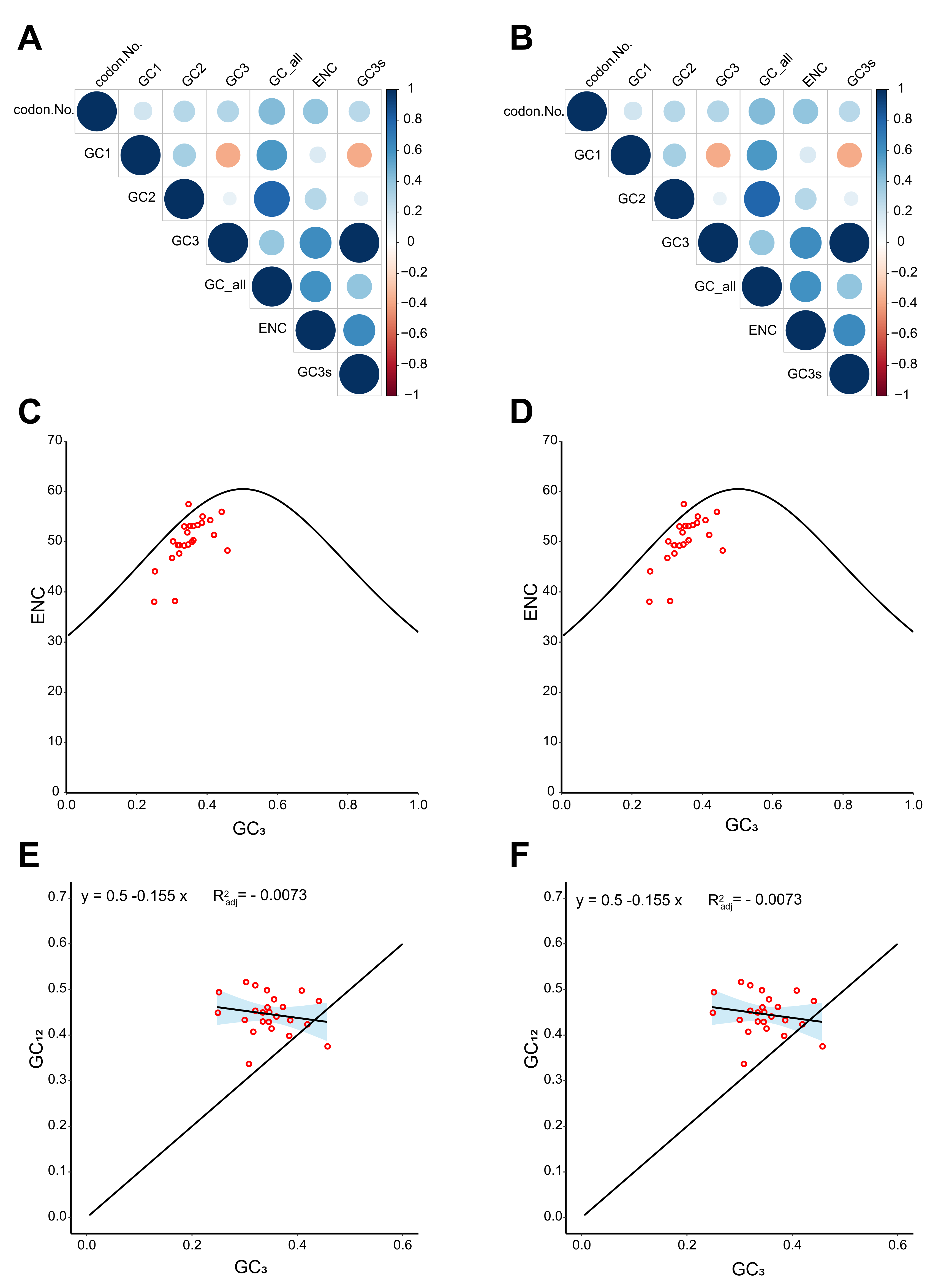


Fig. S1. Analysis of correlation of GC content and ENC value (A), ENC plot (C) and neutrality plot (E) in the *A. heterophyllus* mitochondrial genomes. Correlation of GC content and ENC value (B), ENC plot (D) and neutrality plot (F) in *A. heterophyllus*(R) mitochondrial genomes.


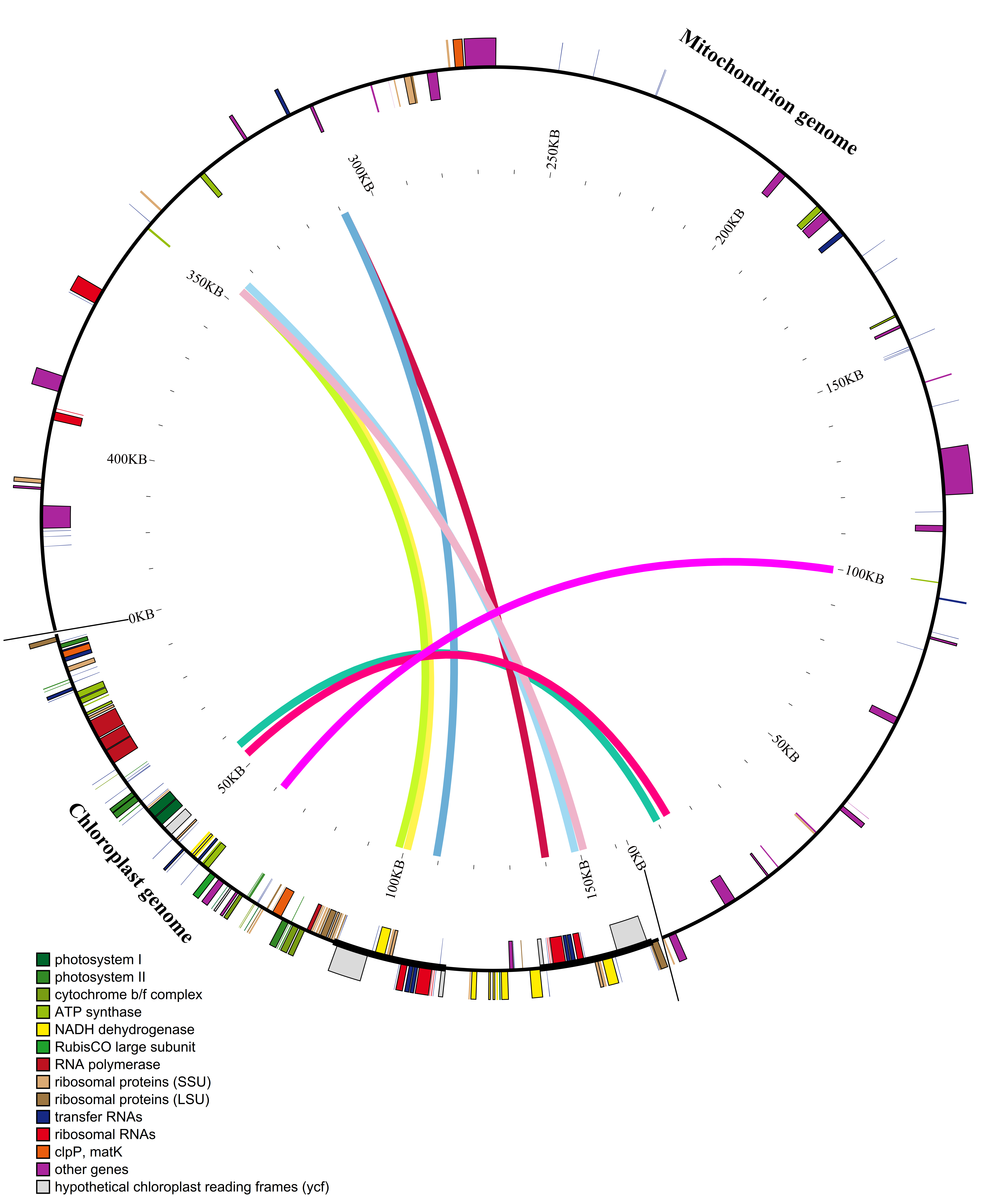


Fig. S2. Locations of the transferred fragments between mitochondrial and chloroplast genomes of *A. heterophyllus*(A) (PQ839731.1, PQ835410.1).


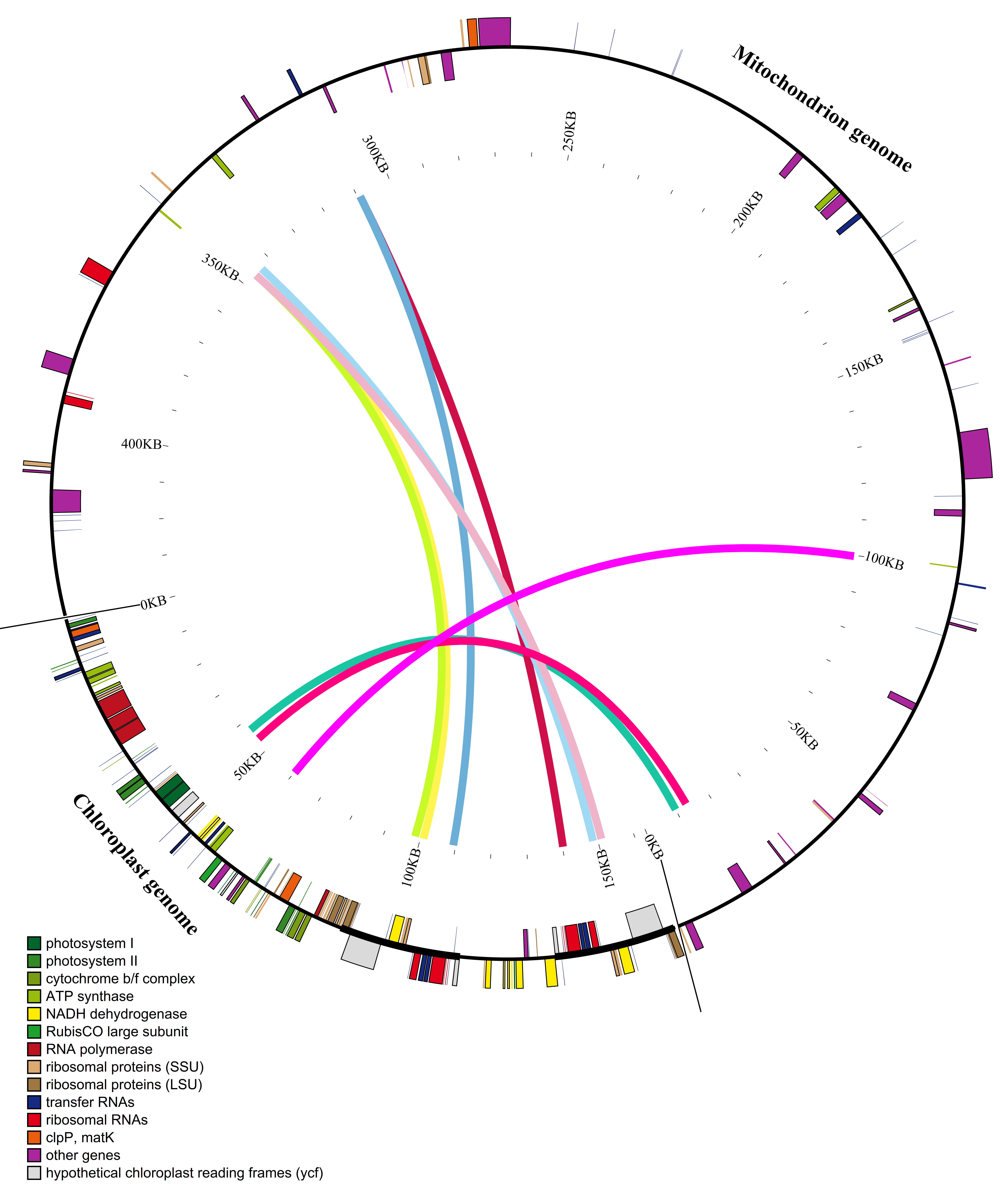


Fig. S3. Locations of the transferred fragments between mitochondrial and chloroplast genomes of *A. heterophyllus*(R) (PQ839730.1, PQ835412.1).


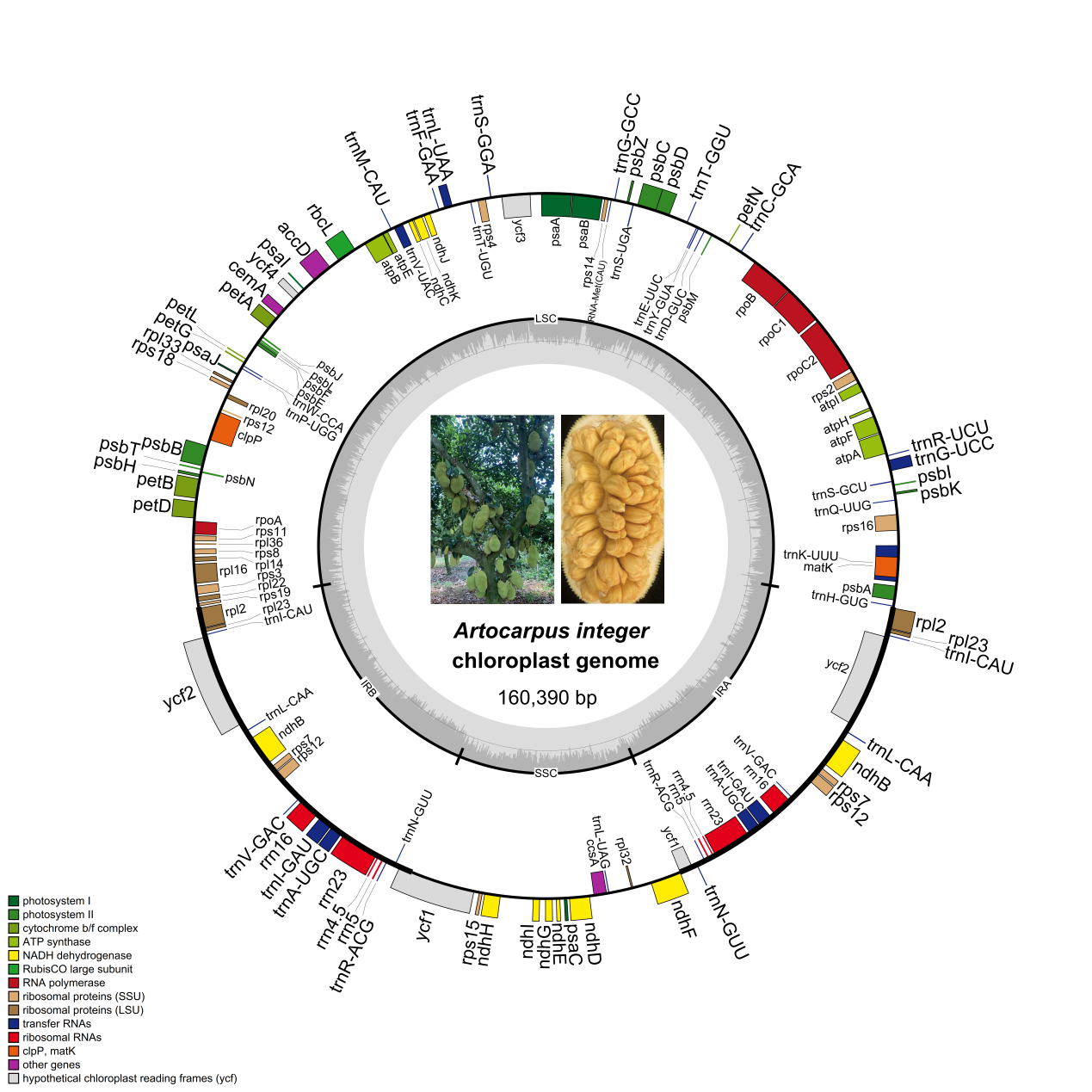


Fig. S4. Circular maps of chloroplast genome in *A. integer*. Genes belonging to different functional groups are shown in different colors in the outermost ring.


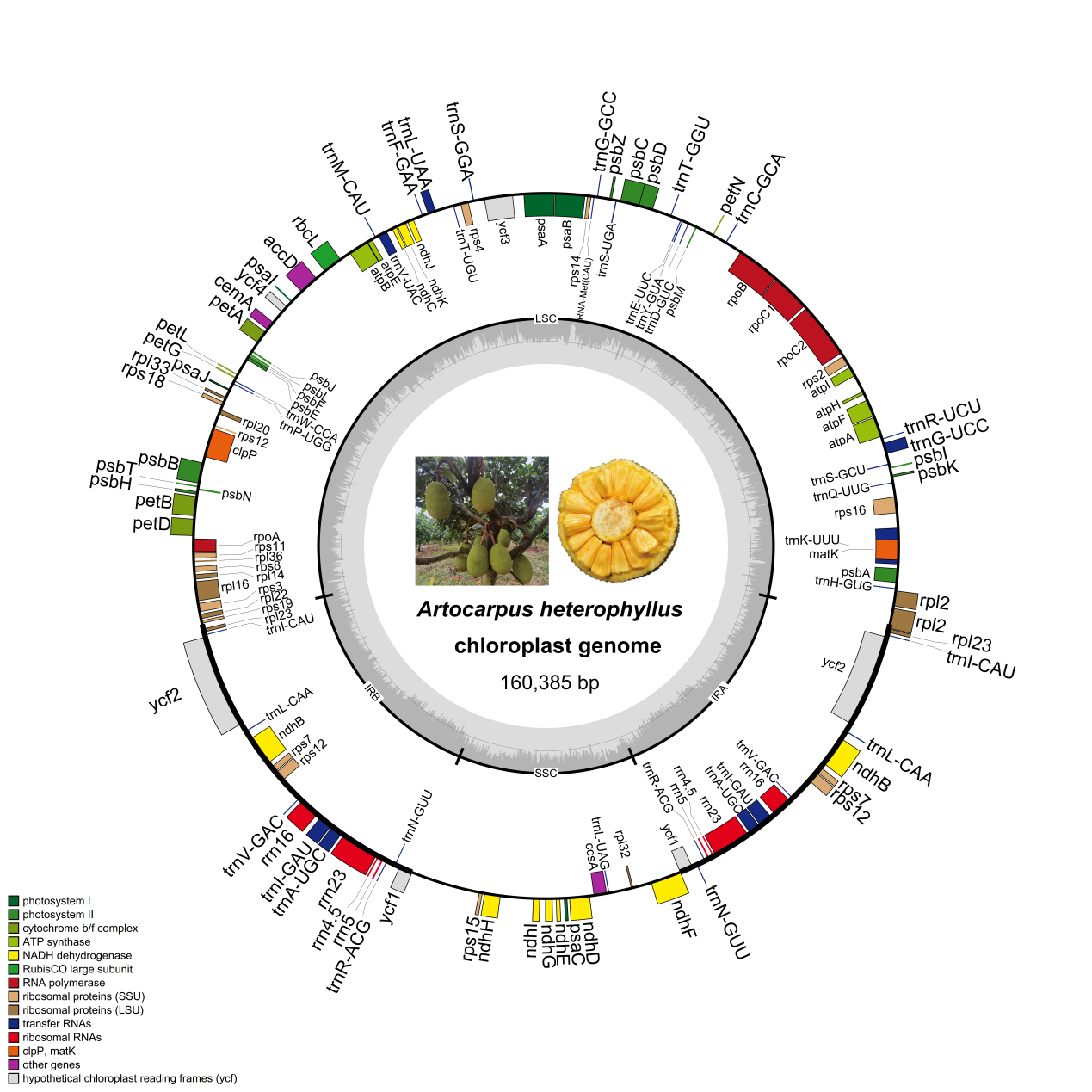


Fig. S5. Circular maps of chloroplast genome in *A. heterophyllus*. Genes belonging to different functional groups are shown in different colors in the outermost ring.


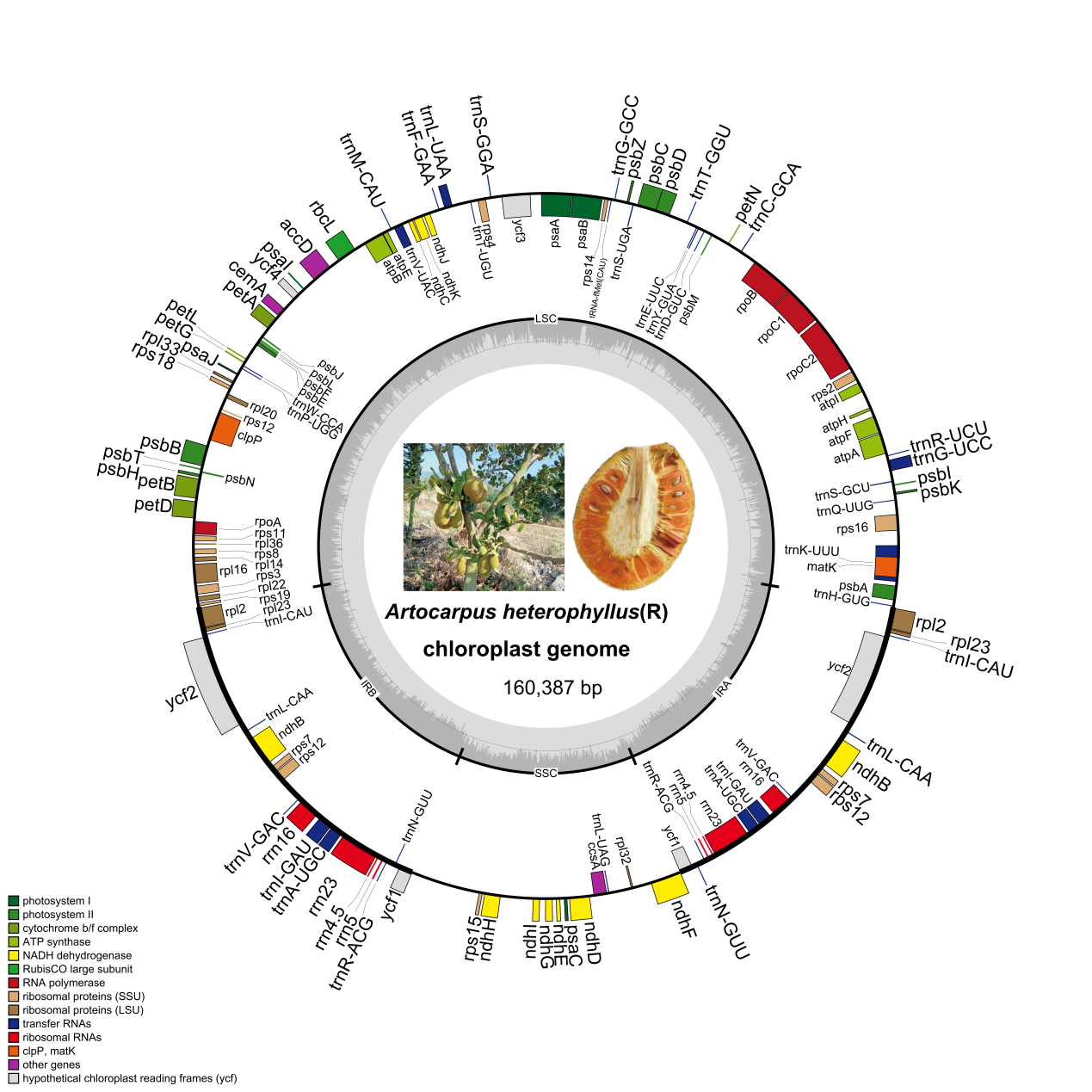


Fig. S6. Circular maps of chloroplast genome in *A. heterophyllus*(R). Genes belonging to different functional groups are shown in different colors in the outermost ring.

| TableS1: The relative synonymous codon usage of each amino acid in *A. integer*. | | | |
| --- | --- | --- | --- |
| Codon | Amino Acid | Number | RSCU |
| CGC | Arg | 49 | 0.61 |
| GUU | Val | 175 | 1.39 |
| UGU | Cys | 75 | 1.24 |
| AAG | Lys | 87 | 0.67 |
| CAA | Gln | 173 | 1.57 |
| GCU | Ala | 213 | 1.66 |
| AAC | Asn | 76 | 0.61 |
| AGA | Arg | 108 | 1.35 |
| CGG | Arg | 53 | 0.66 |
| UCU | Ser | 164 | 1.4 |
| AUA | Ile | 164 | 0.8 |
| CUG | Leu | 79 | 0.53 |
| CCC | Pro | 75 | 0.7 |
| ACA | Thr | 103 | 0.97 |
| UUU | Phe | 309 | 1.17 |
| CCG | Pro | 60 | 0.56 |
| CUC | Leu | 77 | 0.52 |
| GGU | Gly | 183 | 1.35 |
| CCA | Pro | 113 | 1.05 |
| ACG | Thr | 55 | 0.52 |
| AUC | Ile | 157 | 0.77 |
| CUA | Leu | 135 | 0.91 |
| AUG | Met | 228 | 1 |
| GAU | Asp | 180 | 1.43 |
| ACC | Thr | 104 | 0.98 |
| CAC | His | 42 | 0.4 |
| CGA | Arg | 104 | 1.3 |
| UAU | Tyr | 200 | 1.57 |
| AGG | Arg | 53 | 0.66 |
| AGC | Ser | 67 | 0.57 |
| CAG | Gln | 48 | 0.43 |
| AAA | Lys | 173 | 1.33 |
| UAA | end | 13 | 1.56 |
| GUC | Val | 79 | 0.63 |
| CGU | Arg | 113 | 1.41 |
| GCG | Ala | 55 | 0.43 |
| UGC | Cys | 46 | 0.76 |
| AAU | Asn | 175 | 1.39 |
| UGG | Trp | 118 | 1 |
| GCC | Ala | 110 | 0.86 |
| GUG | Val | 108 | 0.86 |
| UCC | Ser | 121 | 1.03 |
| GGG | Gly | 89 | 0.66 |
| UUG | Leu | 172 | 1.16 |
| CCU | Pro | 181 | 1.69 |
| UUC | Phe | 221 | 0.83 |
| GAA | Glu | 232 | 1.45 |
| GGC | Gly | 70 | 0.52 |
| UCG | Ser | 80 | 0.68 |
| CUU | Leu | 188 | 1.27 |
| GAG | Glu | 88 | 0.55 |
| UCA | Ser | 139 | 1.18 |
| AUU | Ile | 293 | 1.43 |
| GGA | Gly | 199 | 1.47 |
| UUA | Leu | 235 | 1.59 |
| ACU | Thr | 162 | 1.53 |
| GAC | Asp | 72 | 0.57 |
| CAU | His | 167 | 1.6 |
| UGA | end | 9 | 1.08 |
| UAC | Tyr | 54 | 0.43 |
| GUA | Val | 141 | 1.12 |
| AGU | Ser | 133 | 1.13 |
| UAG | end | 3 | 0.36 |
| GCA | Ala | 135 | 1.05 |

| Table S2: The collinear blocks longer than 1000 bp between each pair of species | | |
| --- | --- | --- |
|  |  |  |
| Type | Number | total legth |
| *Crataegus pinnatifida* and *Cannabis sativa* | 48 | 97584 |
| *Cannabis sativa* and *Rosa chinensis* | 35 | 69045 |
| *Rosa chinensis* and *Rosa hybrid* | 16 | 267394 |
| *Rosa hybrid* and *Rosa laevigata* | 25 | 237228 |
| *Rosa laevigata* and *Ficus carica* | 31 | 62713 |
| *Ficus carica* and *A. integer* | 60 | 158497 |
| *A. integer* and *A. heterophyllus* (R) | 5 | 472875 |
| *A. heterophyllus* (R) and *A. heterophyllus* | 5 | 472880 |
| *A. heterophyllus* and *Morus notabilis* | 53 | 161344 |
| *Morus notabilis* and *Hemiptelea_davidii* | 52 | 121419 |
| *Hemiptelea_davidii* and *Hippophae tibetana* | 68 | 156306 |
| *Hippophae tibetana* and *Ziziphus jujuba* | 59 | 141223 |

| Table S3. Statistics of RNA editing U(T)-to-C in *A. integer* | | | | | | |
| --- | --- | --- | --- | --- | --- | --- |
|  |  |  |  |  |  |  |
| Gene | Nt pos | AA Pos | Codon pos | Change | Effect | RNA depth(editing/Non-editing) |
| *cob* | 503 | 168 | 2 | T->C | GTG (V) => GCG (A) | '10/2 |
| *ccmFn* | 962 | 321 | 2 | T->C | GTT (V) => ACC (T) | '3/0 |
| *ccmFn* | 963 | 321 | 3 | T->C | GTT (V) => ACC (T) | '3/0 |
| *ccmFn* | 1050 | 350 | 3 | T->C | AAT (N) => AAC (N) | '3/0 |
| *nad*7 | 544 | 182 | 1 | T->C | TTT (F) => CTT (L) | '4/9 |
| *matR* | 1471 | 491 | 1 | T->C | TAT (Y) => CAT (H) | '2/2 |
| *rpl*16 | 114 | 38 | 3 | T->C | TGT (C) => TGC (C) | '6/0 |
| *rps*19 | 147 | 49 | 3 | T->C | ATT (I) => ATC (I) | '5/2 |
| *rps*19 | 195 | 65 | 3 | T->C | GTT (V) => GTC (V) | '5/4 |
| *rps*19 | 224 | 75 | 2 | T->C | ATA (I) => ACA (T) | '3/4 |
| *rps*7_2 | 140 | 47 | 2 | T->C | ATG (M) => ACG (T) | '4/18 |

| Table S4. Statistics of RNA editing U(T)-to-C in *A. heterophyllus*(R) | | | | | | |
| --- | --- | --- | --- | --- | --- | --- |
| Gene | Nt pos | AA Pos | Codon pos | Change | Effect | RNA depth(editing/Non-editing) |
| *rpl*16 | 114 | 38 | 3 | T->C | TGT (C) => TGC (C) | '4/9 |
| *rps*19 | 147 | 49 | 3 | T->C | ATT (I) => ATC (I) | '2/0 |
| *rps*19 | 168 | 56 | 3 | T->C | GTT (V) => ATC (I) | '2/0 |
| *rps*19 | 195 | 65 | 3 | T->C | GTT (V) => GTC (V) | '2/0 |
| *rps*19 | 224 | 75 | 2 | T->C | ATA (I) => ACA (T) | '2/0 |

| Table S5. Statistics of RNA editing U(T)-to-C in *A. heterophyllus* | | | | | | |
| --- | --- | --- | --- | --- | --- | --- |
| Gene | Nt pos | AA Pos | Codon pos | Change | Effect | RNA depth (editing/ Non-editing) |
| *cob* | 503 | 168 | 2 | T->C | GTG (V) => GCG (A) | '51/53 |

| Table S6. Statistics of the Chloroplast genome of three *Artocarpus*species | | | |
| --- | --- | --- | --- |
|  |  |  |  |
| Type | *A. integer* | *A. heterophyllus* | *A. heterophyllus*(R) |
| Total length | 160390 bp | 160387 bp | 160387 bp |
| LSC | 89078 bp | 89075 bp | 89077bp |
| SSC | 19894 bp | 19896 bp | 19894 bp |
| IR | 25709 bp | 25708 bp | 25708 bp |
| GC% | 36.05% | 36.06% | 36.06% |
| Total | 129 | 130 | 129 |
| PCGs | 84 | 85 | 84 |
| rRNA | 8 | 8 | 8 |
| tRNA | 37 | 37 | 37 |
